# Polyvinylpyrrolidone–Alginate Film Barriers for Abdominal Surgery: Anti-Adhesion Effect in Murine Model

**DOI:** 10.3390/ma16165532

**Published:** 2023-08-09

**Authors:** Anna A. Forysenkova, Mariya V. Konovalova, Inna V. Fadeeva, Olga S. Antonova, Olga D. Kotsareva, Tatiana K. Slonskaya, Julietta V. Rau, Elena V. Svirshchevskaya

**Affiliations:** 1 Baikov Institute of Metallurgy and Material Science RAS, Leninsky Av., Build. 49, 119334 Moscow, Russia; aforysenkova@gmail.com (A.A.F.); fadeeva_inna@mail.ru (I.V.F.); osantonova@yandex.ru (O.S.A.); 2 Shemyakin-Ovchinnikov Institute of Bioorganic Chemistry RAS, Miklukho-Maclay Str., Build. 16/10b, 117997 Moscow, Russia; mariya.v.konovalova@gmail.com (M.V.K.); olga.kotsareva@gmail.com (O.D.K.); 3 Department of Analytical, Physical and Colloid Chemistry, I.M. Sechenov First Moscow State Medical University, Trubetskaya Str., Build. 8/2, 119991 Moscow, Russia; slonskaya_t_k@staff.sechenov.ru; 4 Istituto di Struttura della Materia, Consiglio Nazionale delle Ricerche (ISM-CNR), Via del Fosso del Cavaliere, 100, 00133 Rome, Italy

**Keywords:** polyvinylpyrrolidone, alginate, anti-adhesion materials, peritoneal surgery, gene expression

## Abstract

Surgical operations on the peritoneum are often associated with the formation of adhesions, which can interfere with the normal functioning of the internal organs. The effectiveness of existing barrier materials is relatively low. In this work, the effectiveness of soluble alginate–polyvinylpyrrolidone (PVP-Alg) and non-soluble Ca ion cross-linked (PVP-Alg-Ca) films in preventing these adhesions was evaluated. Experiments in vivo were performed on mice via mechanical injury to the adjacent peritoneum wall and the caecum, followed by the application of PVP-Alg or PVP-Alg-Ca films to the injured area. After 7 days, samples from the peritoneal wall and caecum were analyzed using histology and quantitative polymerase chain reaction (qPCR). It was shown that the expression of genes responsible for adhesion formation in the caecum in the PVP-Alg group was comparable to that in the control group, while in the PVP-Alg-Ca group, it increased by 5–10 times. These results were consistent with the histology: in the PVP-Alg group, the adhesions did not form, while in the PVP-Alg-Ca group, the adhesions corresponded to five points on the adhesion scale. Therefore, the formation of intraperitoneal adhesions can be effectively prevented by non-crosslinked, biodegradable PVP-Alg films, whereas cross-linked, not biodegradable PVP-Alg-Ca films cause inflammation and adhesion formation.

## 1. Introduction

Intraperitoneal adhesions (IPAs) form in 20–80% of cases of surgical operations [1]. IPAs are more common in men after peritoneal surgery (2:1, men to women) and in women after appendectomy (3:1, women to men). The main causative factor of the formation of IPAs is the interaction between the abdominal mucosa and the membranes of organs. The risk factors include blood clots in the peritoneum, peritonitis, the size of the damaged area upon interventions, and co-morbidity diseases. The adhesive process is also influenced by such factors as decreases in intestinal tone during the postoperative period or a significant increase in intraperitoneal pressure. Re-operations increase the risk of adhesive disease by 20% after the first intervention and up to 50% after the fifth surgery. The prevention and treatment of adhesions after peritoneal surgery, as well as after gynecological and cardiac surgery, are particularly challenging tasks [2].

At present, the main method of preventing adhesions is the application of chemical (pharmacological) or physical (mechanical) barriers [2]. Physical barriers are preferable, as they do not induce bleeding or immunosuppression [3].

Existing physical barrier materials are based on natural and synthetic polymers used in the form of gels or films [3,4]. There are a number of requirements for barrier materials, such as biocompatibility, the absence of toxic by-products, and the ability to biodegrade or to be bioabsorbed [5,6,7]. In addition, the material should not damage the surrounding soft tissues but at the same time should be viscous enough and last for ease of handling during the operation; it should also not require additional means of fixation [2]. Ideally, such materials should not only prevent the formation of adhesions, but also have some healing factors, such as the stimulation of tissue regeneration, anti-inflammatory action, and antibacterial effects [2,5,8]. 

Natural polymers have excellent biocompatibility and are widely available [3,9]. The most common natural polymers are polysaccharides–chitosan, alginate (Alg), cellulose, hyaluronic acid (HA), pectin, and starch [3]. Some polysaccharides can be rapidly decomposed by endogenous enzymes in vivo [3,10]. In particular, HA can be decomposed by hyaluronidase [11]. The rate of biodegradation can be controlled by gamma irradiation (for HA and its derivatives) or ionic cross-linking (for Alg) [2,12,13].

Synthetic polymers are preferred because their manufacturing allows for the reproducibility of the properties. Synthetic polymers such as polyethylene glycol (PEG), lactic acid derivatives (polylactide, PLA) [14], polycaprolactone (PCL) [15], polyvinyl alcohol (PVA) [16], and polyvinylpyrrolidone (PVP) are already used in clinics [3,4]. These polymers are biocompatible, bioinert (PEG, PVA, PVP), and non-immunogenic [17]. PEG, PVA, and PVP cannot be degraded with specific enzymes, which means they do not emit toxic by-products [18].

PVP and Alg look promising for the development of anti-adhesion materials. The choice of PVP and Alg was determined by their hydrophilic nature and low cell adhesion [19]. Alg has a negative charge, which plays a role in its anti-adhesion effect [20,21]. In addition, Alg is widely used in pharmaceuticals and food production [13]. PVP, a non-toxic, non-immunogenic hemocompatible polymer [22], has been used as a blood plasma substitute [23]. Currently, PVP is widely used to create various forms of drugs and implants for controlled drug delivery [24]. When administered orally, low-molecular-weight PVP is easily excreted by the kidneys without side effects [23].

Previously, we studied the physical–chemical properties of PVP-alginate film materials: mechanical strength, swelling, and biocompatibility in vitro [25,26]. In the present work, attention is focused on the investigation of the in vivo behavior of soluble PVP-Alg and insoluble Ca-ion-crosslinked PVP-Alg-Ca films. The microstructure of the PVP-Alg and PVP-Alg-Ca films and the morphology of mouse fibroblasts on the surface of the insoluble-film PVP-Alg-Ca were studied, and the solubility of the PVP-Alg-Ca film in water was measured. An in vivo study of the anti-adhesion activity of the above-mentioned films was conducted via intraperitoneal surgery in mice followed by histological and quantitative polymerase chain reaction (qPCR) tests. Based on the obtained data from the visual evaluation of adhesions according to a five-point scale of adhesion formation, as well as on the histology and analysis of gene expression, conclusions were drawn regarding the anti-adhesion activity of the proposed films and the possibility of their use in abdominal surgery.

## 2. Materials and Methods

### 2.1. PVP-Alg Film and PVP-Alg-Ca Film Preparation

**Figure 1:**
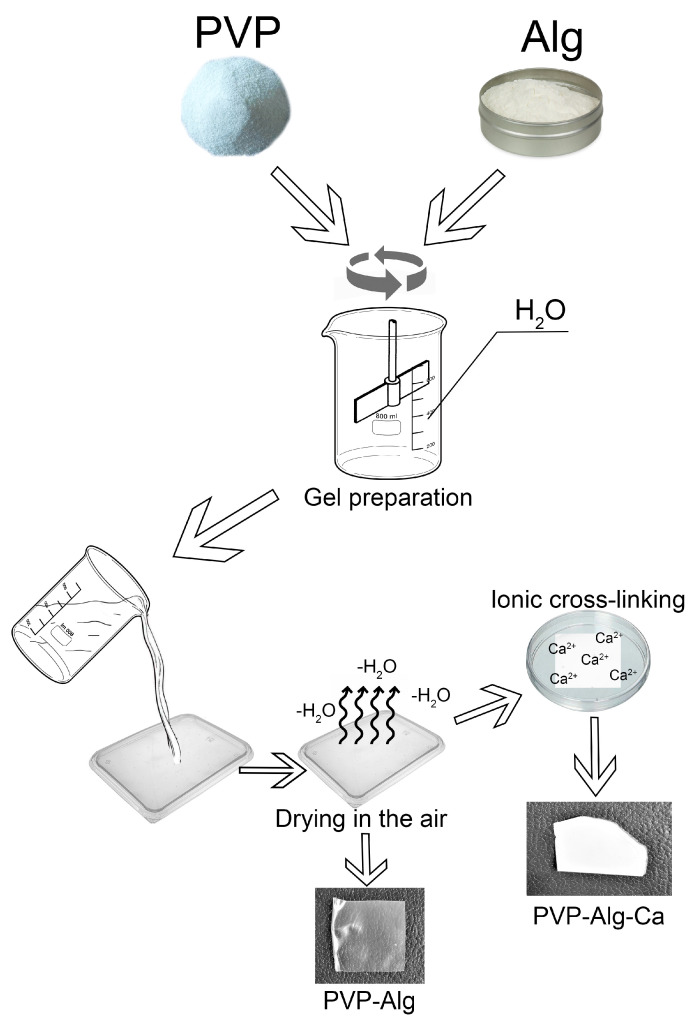
Scheme of PVP-Alg film and PVP-Alg-Ca film preparation.

PVP (360 kDa; Sigma-Aldrich, Saint-Quentin Fallavier, France) and alginate (Alg) (Ingrid, food, Voronezh, Russia) were dissolved in deionized water at a ratio of 1:1 to obtain a 2.5% solution and mixed at 700 rpm until homogeneity was achieved. The resulting gel was placed in polypropylene pads and dried in the air until dry films of PVP-Alg were obtained. To prepare PVP-Alg-Ca films, the dried PVP-Alg films were immersed for 5 min in 50 mL of 0.5 M CaCl_2_ solution for partial crosslinking with calcium ions [27] and dried at 40 °C. As a result of drying, hard brittle PVP-Alg-Ca films were formed. The preparation scheme is shown in Figure 1.

### 2.2. Scanning Electron Microscopy (SEM) Investigation 

A scanning electron microscope, Tescan VEGA3 (Tescan, Brno, Czech Republic), was used to study the microstructure of the PVP-Alg and PVP-Alg-Ca films before and after soaking, as well as the morphology of attached cells on the surface. The secondary electron mode at an accelerated voltage of 20 kV was used. The Q150R Plus rotary pump spraying system was used to pre-spray gold onto the surface of the dried samples (Quorum Technologies Ltd., Lewes, Great Britain).

### 2.3. Investigation of PVP-Alg-Ca Film Solubility 

To evaluate the solubility of the PVP-Alg-Ca films, 0.5 g of the samples was soaked in 50 mL of deionized water at 37 °C for 1, 10, 28, and 42 days. To calculate the mass loss, the samples were dried at 40 °C and then weighed in a desiccator.

### 2.4. Investigation of Fibroblast L929 Attachment

For a qualitative assessment of cell attachment to the insoluble PVP-Alg-Ca material, SEM images of mouse fibroblast L929 (PanEco, Moscow, Russia) on the surface of the samples were also obtained. To achieve this, fragments of PVP-Alg-Ca films of 1 cm in diameter were placed in a 24-well plate, and a suspension of fibroblasts in a growth medium with a seeding density of 3000 cells/cm^2^ was introduced; afterward, the 24-well plate was placed in a CO_2_ incubator for 24 h. The fibroblasts were fixed with a 2.5% solution of glutaraldehyde for 30 min at room temperature. The samples were successively washed with ethanol solutions (50%, 75%, 80%, 90%, and 100%) for 5 min at each concentration. Finally, the samples were airdried.

### 2.5. Anti-Adhesive Activity of PVP-Alg and PVP-Alg-Ca Films in Murine Model 

CD1 male mice (40 ± 5 g) were taken from the laboratory of the “Pushchino” animal nursery in the Moscow region. All animals had unhindered access to food and water and were kept in conventional conditions. CD1 mice were anesthetized with xylazine hydrochloride (Alfasan International B.V., Woerden, The Netherlands) (20 mg/mL) at a dose of 5 mg/kg and telazol (Zoetis, Parsippany-Troy Hills, NJ, USA) (100 mg/mL) at 70 mg/kg. The animals were divided into 5 groups: sham-operated mice; control mice operated on without barrier films; and mice operated on using PVP-Alg dry films, using moisturized and swelled PVP-Alg films (hereinafter «gels») and PVP-Alg-Ca films. Earlier we showed that the adhesion scores of CD1 mice increased from the 2nd to 4th day to the 7th day post-operation [28]. Therefore, to evaluate the formation of adhesions, mice were withdrawn from the experiment after 7 days.

The aseptic technique was used throughout the experimental period. The fur on the belly was removed with a depilator. The skin surface was sterilized with an iodine solution. An incision about 1 cm long was made with eye scissors along the midline of the anterior wall of the peritoneal cavity. The caecum was extracted with tweezers and wrapped with a sterile gauze cloth and a 0.5 × 0.5 cm surface area was fixed and rubbed using a sterile abrasive material until spot bleeding was achieved. A section of the muscle layer of 0.5 × 0.5 cm on the lateral wall of the peritoneal cavity adjacent to the caecum was also rubbed until spot bleeding was achieved. In the experimental groups, the following films were applied to the injured surface: PVP-Alg gel, PVP-Alg dry film, and PVP-Alg-Ca film, all in the form of circles of 1 cm in diameter. The injured caecum was placed back in the peritoneal cavity near the injured peritoneal wall. All mice recovered from anesthesia and were active a day after the surgical operation. The animals were euthanized after the operation. The peritoneal cavity was opened with a U-shaped incision, and the severity of adhesions was evaluated on a five-point scale of adhesion formation (Table 1) [29].

### 2.6. Analysis of Gene Expression

Tissue samples (injured peritoneal wall and caecum, ≈100 mg) taken at autopsy were homogenized in 1 mL of ExtractRNA solution (EuroGen, Moscow, Russia). RNA was extracted as recommended by the manufacturer. The resulting RNA was dissolved in the required volume of RNase-free water. Genomic DNA was removed using a kit (Thermo Scientific, Waltham, MA, USA). cDNA was obtained from the isolated RNA using a commercial Reverse Transcriptase M-MuLV–RH kit (Biolabmix, Novosibirsk, Russia). The resulting cDNA was stored at −20 °C until a quantitative real-time polymerase chain reaction (qPCR) was performed using a BioMaster HS-qPCR SYBR Blue (2×) kit (Biolabmix, Novosibirsk, Russia). The reaction was carried out in a volume of 20 µL using specific primers. The list of primers is given in Table 2.

Cycle threshold (Ct) values for the target genes were normalized to the Ct value for the housekeeping gene β-actin. The relative expression of the target genes was calculated by comparing the Ct value for the tissue from experimental mice to intact tissue. Data are presented as fold changes in mRNA levels in the tissue of experimental mice compared with the intact ones or to the surgery mice. To carry out the reaction, an amplifier using real-time detection with CFX Connect (Bio-Rad, Hercules, CA, USA) was used. The results of the qPCR were processed using the CFX Manager program (Bio-Rad, Hercules, CA, USA). The expression of each gene was analyzed in three replicates.

### 2.7. Histology

Tissue samples of the peritoneal wall and caecum were treated with paraformaldehyde (4%) and filled with paraffin. The samples were cut into slices with a thickness of 4 µm using a Leica RM 2145 RTS cryomicrotome (Leica Biosystems, Nussloch, Germany). After the removal of paraffin from the tissue sections, they were stained with hematoxylin-eosin (H&E) or a commercial Trichome Mason stain (Biovitrum, Novosibirsk, Russia) according to the manufacturer’s protocol. Then, the sections were dehydrated, lightened, and enclosed under a cover glass using a histological medium: Consul-Mount (Thermo-Scientific, Waltham, MA, USA). The stained samples were examined using a Zeiss Primo Starlight microscope (Carl Zeiss, Oberkochen, Germany).

### 2.8. Statistical Analysis

Graphs were created using MS Excel. The data are represented as mean ± standard error of the mean for at least three independent experiments or as one representative experiment from three. Statistical analysis was performed using Student’s *t*-test. Significance levels of *p* < 0.05 were considered statistically reliable.

## 3. Results

### 3.1. Microstructure, Cells Attachment, and Solubility of PVP-Alg and PVP-Alg-Ca Films

**Figure 2:**
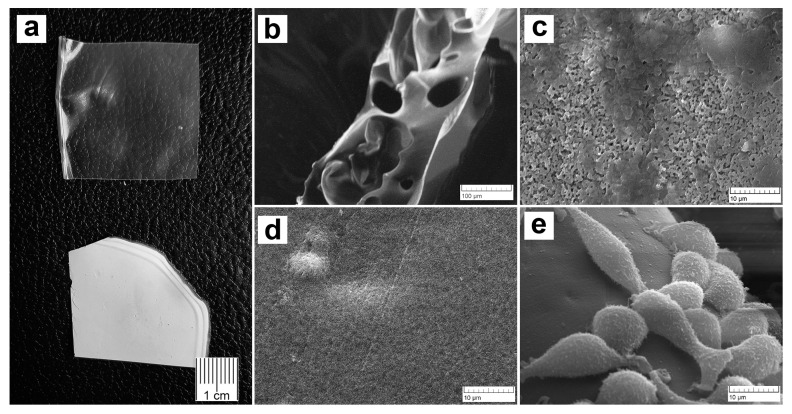
Characteristics of PVP-Alg and PVP-Alg-Ca films. PVP-Alg (**a**, upper) and PVP-Alg-Ca (**a**, lower) dried films; SEM images of porous PVP-Alg dry film (**b**); dense porous upper (**c**) and smooth nonporous lower (**d**) sides of the PVP-Alg-Ca film; SEM image of murine fibroblasts L929 incubated on the smooth side of PVP-Alg-Ca film for 24 h (**e**).

Microphotographs of the films are shown in Figure 2. The PVP-Alg dry films are transparent, colorless, and glossy, with large pores with thin walls (Figure 2a, upper and b). The PVP-Alg-Ca films are opaque white and matte (Figure 2a, lower). The PVP-Alg-Ca films have different upper and lower surfaces. The upper side of the PVP-Alg-Ca film is rough and porous with some smooth areas (Figure 2b). The side of the PVP-Alg-Ca film adjacent to the pad is smooth with no pores (Figure 2c). This may be due to the redistribution of PVP from the immersion of the film in the CaCl_2_ solution. The morphology of L929 fibroblasts on the partially smooth surface of the PVP-Alg-Ca is shown in Figure 2d. However, the cells do not form a monolayer on the surface of the film, which indicates their low adhesion to the material. This behavior of cells on the surfaces of crosslinked alginate films was observed in ref. [30]. Previously, the absence of platelet adhesion to the PVP surface and a decrease in fibrinogen adsorption were shown in [31]. A similar effect was also observed with respect to bacteria on the surface of the PVP-Alg gels [32].

**Figure 3:**
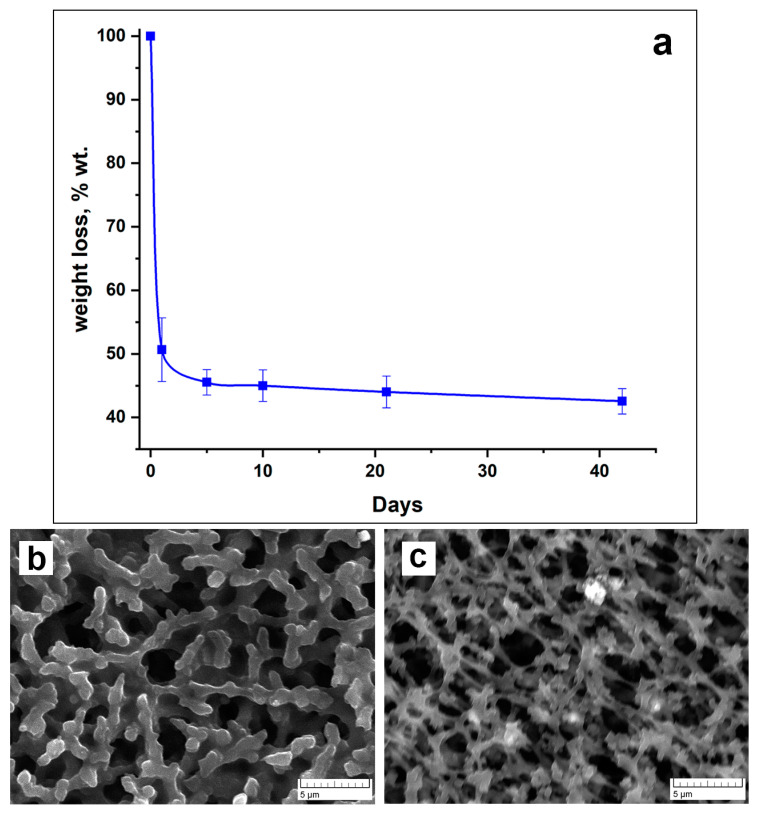
PVP-Alg-Ca dissolution characteristics: dissolution curve (**a**) (the error bar is the arithmetic mean); SEM of PVP-Alg-Ca surface after 24 h (**b**); SEM of PVP-Alg-Ca surface after 10 days (**c**).

To evaluate the possibility of PVP-Alg-Ca biodegradation, we carried out a study on the solubility of PVP-Alg-Ca in deionized water. As can be observed from Figure 3a, the samples quickly lose weight by up to ≈50% during the first 24 h and then reach a plateau during the next 42 days (Figure 3a). This is a result of a crosslinked insoluble alginate “grid” formation, with soluble PVP released from the film during the first few hours, as shown in ref. [32]. After 24 h, the sample becomes more porous, and the polymer acquires a dendritic structure (Figure 3b). The micrographs after 10 days of immersion show that the PVP-Alg-Ca film is still solid (Figure 3c).

### 3.2. Prevention of Adhesion Formation by PVP-Alg and PVP-Alg-Ca Films

It is known that Alg is degraded by alginate lyase, which is only found in algae, marine invertebrates, and microorganisms, from which Alg is extracted [33,34]. Mammals do not have a specific enzyme through which Alg can be degraded. However, any exogenous materials can be eliminated by unspecific mechanisms, such as macrophages. In the case of enzymatically nondegradable biopolymers, in order to be eliminated by macrophages, the biopolymers should easily dissociate into small particles comparable to the size of microorganisms. This property of anti-adhesion materials is very important.

**Figure 4:**
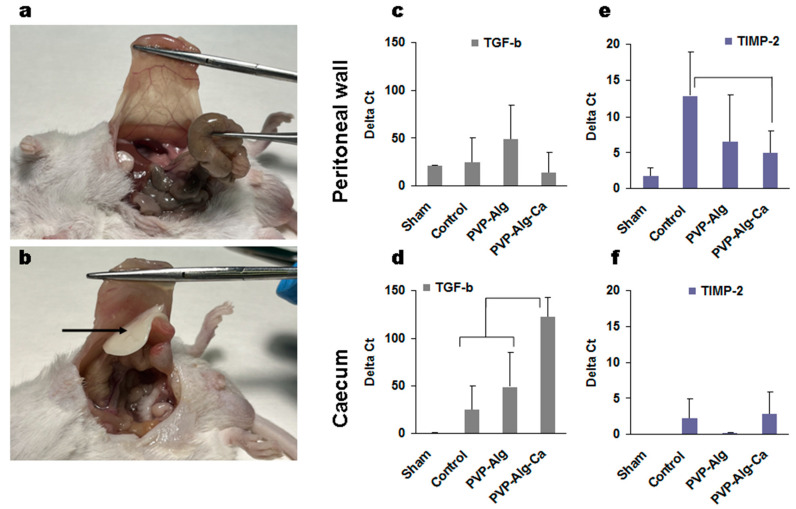
Surgery site overview and gene expression of the peritoneal wall and caecum cells from the injured sites. (**a**,**b**) Overview of the peritoneal cavity of PVP-Alg (**a**) and PVP-Alg-Ca (**b**) mice. Black arrow shows PVP-Alg-Ca film. (**c**–**f**) The expression of transforming growth factor β (TGF-β) (**c**,**d**) and tissue matrix metalloprotease inhibitor 2 (TIMP-2) (**e**,**f**) in the sham group, control group, PVP-Alg gel group, and PVP-Alg-Ca film groups. Significant differences are shown as square brackets. The error bar is the mean standard deviation, *p* < 0.05.

Both PVP-Alg dry films and gels completely prevented the formation of adhesions (Table 3). It should be noted that the PVP-Alg dry films immediately gelled when applied to the tissue and were not detected in the cavity on the 7th day (Figure 4a). Contrary to the PVP-Alg material, the crosslinked PVP-Alg-Ca films did not degrade over 7 days. The crosslinked PVP-Alg-Ca films in the peritoneal cavity were found to be almost unchanged (Figure 4b, arrow).

### 3.3. Gene Expression Induced by Model Surgery 

**Figure 5:**
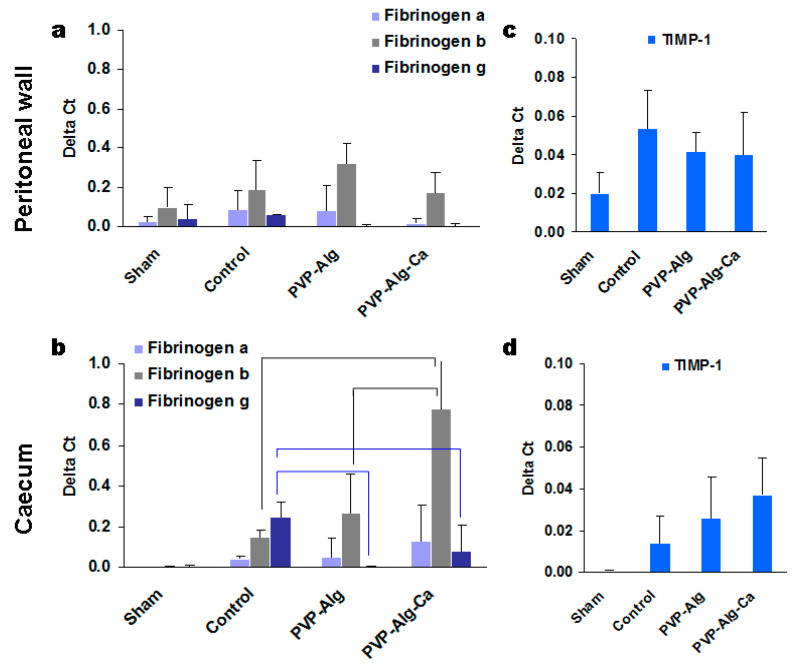
Gene expressions of the peritoneal wall and caecum cells from the injured sites. The expression of fibrinogens αβγ (**a**,**b**) and TIMP-1 (**c**,**d**) in sham, control model surgery, PVP-Alg-gel-treated mice, and PVP-Alg-Ca-film-treated mice in the peritoneal wall (**a**,**c**) and caecum (**b**,**d**) tissues. Significant differences are shown with brackets. The error bar is the mean standard deviation, *p* < 0.05.

The tissue samples from the injured sites of the peritoneal wall and caecum were used to extract RNA for the analysis. Since a sham operation can also induce some changes in gene expression, the effect of the tissue abrasion was compared with the sham-operated mice separately in the peritoneal wall and caecum cells. The expression of TGF-β and TIMP-2 was higher than that of the housekeeping gene actin-β (Figure 4c–f). High expression was also found in the fibrinogen system (Figure 5a,b). Among 20 genes tested, the expressions of hyaluronidase, MMP-1, MMP-2, MMP-3, MMP-5, MMP-7, MMP-8, MMP-10, tropoelastin, and collagen I were neglectable and are not shown.

#### 3.3.1. Transforming Growth Factor β

The main gene is most likely TGF-β since its expression was the highest. When evaluated using ΔCt, its expression in some samples was 50–100 times higher than that of actin-β used as a housekeeping gene (Figure 4c,d). The expression of TGF-β in the peritoneal wall was comparable (*p* > 0.05) between all groups and, consequently, was not specific to the formation of the adhesions.

In contrast to the peritoneal wall cells, the caecum cells responded with a significant increase in TGF-β expression compared with the control group. The crosslinked insoluble PVP-Alg-Ca films increased the TGF-β expression in comparison with the model control and PVP-Alg gel (Figure 4d).

The second highly expressed gene (up to 10–12 ΔCt) was TIMP-2, which was identified mainly in the peritoneal wall cells (Figure 4e,f). The PVP-Alg-Ca film decreased the TIMP-2 expression, which can be related to adhesion formation. At the same time, both the PVP-Alg gel and film also decreased its expression (not statistically significant), so the role of TIMP-2 is still questionable. Both materials, PVP-Alg and PP-Alg-Ca, may decrease its expression, with the difference that the insoluble PVP-Alg-Ca film cannot prevent adhesion formation for other reasons.

#### 3.3.2. Fibrinogens α, β, and γ

The main role of fibrinogen is to induce clot formation after the conversion of thrombin to fibrin. Fibrinogen is synthesized mostly in the liver and released in the blood. Differentiated intestinal epithelial cells also constitutively express fibrinogen [35]. The role of different types of fibrinogens in adhesive disease is not well known. In most cases of wound healing, fibrin is removed, but incomplete removal can lead to the development of various disorders, including the formation of adhesions. An analysis of fibrinogen gene expression demonstrated that all three isoforms increased mostly in the intestinal epithelium but not in the peritoneal wall cells in the control mice compared with the sham-operated ones (Figure 5a,b). There was no difference in fibrinogen α expression between the groups. The aggravation of inflammation in the case of the PVP-Alg-Ca film samples was associated with an increase in fibrinogen β (black brackets), while the expression of fibrinogen γ decreased (blue brackets) in mice with both types of the barrier material (PVP-Alg and PVP-Alg-Ca).

TIMP-1 is another type of tissue metalloprotease inhibitor. Its increased expression was found in both the peritoneal wall and caecum cells compared with the sham samples; however, there were no significant differences between the control and barrier group mice (Figure 5c,d). It was concluded that the involvement of TIMP-1 in adhesion formation is unlikely.

#### 3.3.3. Fibrinolytic Systems tPA and PAI-1

**Figure 6:**
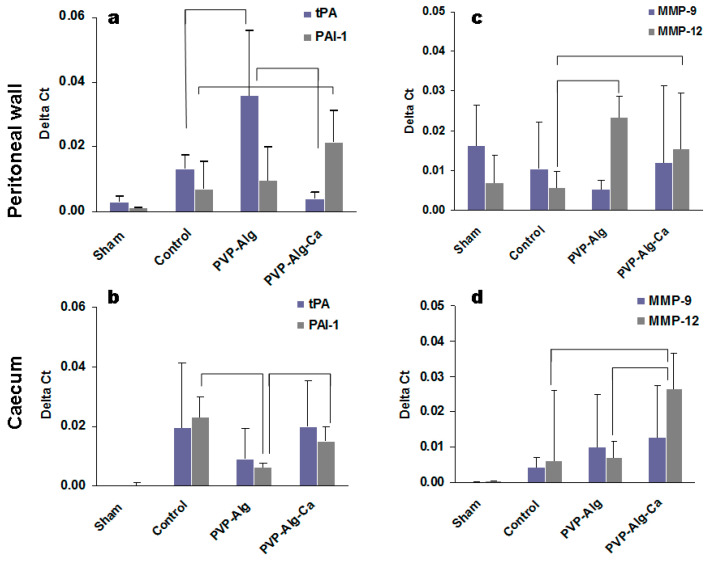
Gene expressions of the peritoneal wall and caecum cells from the injured sites. The expressions of tPA and PAI-1 (**a**,**b**) and MMP-9 and MMP-12 (**c**,**d**) in the sham and control model. Significant differences are shown with brackets. The error bar is the mean standard deviation, *p* < 0.05.

The balance between tissue plasminogen activator tPA and its inhibitor PAI-1 is important for blood clotting and fibrin degradation. Both the peritoneal wall and caecum cells increased tPA and PAI-1 gene expression in the control mice in comparison with the sham-operated mice (Figure 6a,b). The ratios of tPA/PAI-1 in the caecum tissue did not change in different groups; however, the expression of the PAI-1 was lower in the caeca of mice in the presence of the PVP-Alg gel (Figure 6b, brackets). The response in the peritoneal tissue was more informative: the tPA/PAI-1 ratio for the PVP-Alg gel was 3.6 compared with 0.2 in the PVP-Alg-Ca group and 1.9 in the control group.

#### 3.3.4. Matrix Metalloproteinases

PAI-1 also inhibits the activity of matrix metalloproteinases (MMP). Among the many MMPs tested, the only reliable expression was found in the MMP-9 and MMP-12 genes [34,35]. A decrease in MMP-9 expression has been found in patients with intrauterine adhesions [36]. In this work, the results obtained from the peritoneal wall supported the decrease in MMP-9 expression; however, there were no significant differences between the PVP-Alg, PVP-Alg-Ca, and control groups (Figure 6c). At the same time, an increase in MMP-9 expression was found in the epithelial caecum cells in comparison with the sham group, without the differences between the experimental groups.

MMP-12 is a unique protease also known as macrophage elastase, which is produced by macrophages. It is involved in some types of fibrosis [37]. The removal of polymers from the peritoneal cavity is realized by macrophages. MMP-12 could be produced by both resident tissue macrophages and peritoneal macrophages attached to the injured tissue. A higher MMP-12 expression was found both in the peritoneal wall and in the PVP-Alg and PVP-Alg-Ca groups compared with the sham-operated and control mice (Figure 6c), while a higher expression was identified in the caecum only in the PVP-Alg-Ca group (Figure 6d). The activation of MMP-12 is expected when using exogenous material in the body.

#### 3.3.5. Histology of the Adhesions

**Figure 7:**
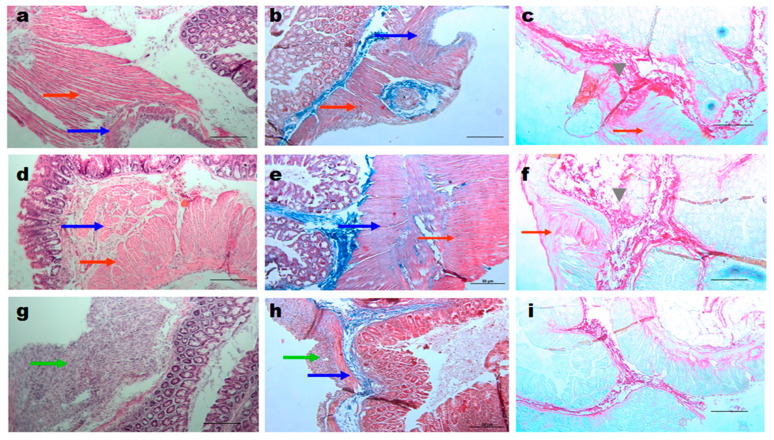
Histology of the injured sites. Samples of control (**a**–**c**), PVP-Alg-Ca (**d**–**f**), and PVP-Alg (**g**–**i**) mice stained with H&E (**a**,**d**,**g**), Trichome Mason (**b**,**e**,**h**), and PicroSirius Red (**c**,**f**,**i**). Blue arrows show caecum muscle layers; red arrows show peritoneal wall muscle layers; green arrows show granular tissue; gray arrowheads show connective tissue formation. Scale bar—50 µm.

In the control groups with abrasions, the caecal fusion tunica muscularis (blue arrows) and peritoneal muscularis externa (red arrows) were observed (Figure 7a–c). The same fusion was also observed in the PVP-Alg-Ca film groups (Figure 7d–f). In contrast to these two groups, the PVL-Alg gel prevented the formation of adhesions and stimulated granular tissue formation (Figure 7g–i, green arrows). The fusion of the muscular layers is an early stage of adhesion formation. Later, they are separated, keeping the connective tissue bonds. The formation of such adhesions was registered after PicroSirius Red staining (Figure 7c,f, gray arrowheads).

#### 3.3.6. Comparison of Gene Expression in Different Groups

**Figure 8:**
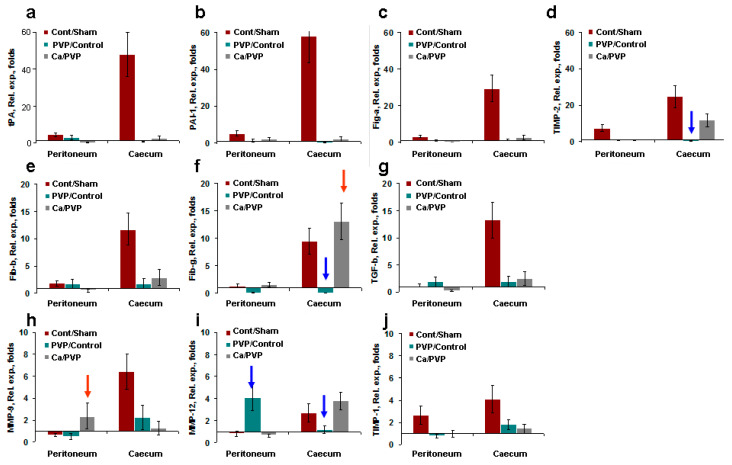
Comparison of the gene expression of PVP-Alg gel and PVP-Alg-Ca film: (**a**) folds of the relative expression of tPA; (**b**)—PAI-1; (**c**)—fibrinogen α; (**d**)—TIMP-2; (**e**)—fibrinogen β; (**f**)—fibrinogen γ; (**g**)—TGF-β; (**h**)—MMP-9; (**i**)—MMP-12; (**j**)—TIMP-1. Columns: ΔCt_control_/ΔCt_sham_ (Cont/Sham, brown columns); Δ_CtPVP-Alg_/ΔCt_control_ (PVP/Control, green columns); and ΔCt_PVP-Alg-Ca_/ΔCt_PVP-Alg_ (Ca/PVP, gray columns) in different genes in the peritoneum and caecum cells. Major protective (blue arrows) and nonprotective (red arrows) effects are shown. The error bar is the mean standard deviation, *p* < 0.05.

The analysis of the gene expression showed the activation of multiple genes induced by the mesothelial and epithelial layer abrasions. To clarify the effects of abrasion versus the sham operation, protection (PVP-Alg) versus control attrition, and aggravation (PVP-Alg-Ca) versus protection (PVP-Alg), relative gene expression folds were calculated using the following formulas: folds = ΔCt_control_/ΔCt_sham_ (brown columns); Δ_CtPVP-Alg_/ΔCt_control_ (PVP/control, green columns); and ΔCt_PVP-Alg-Ca_/ΔCt_PVP-Alg_ (Ca/PVP, gray columns) (see Figure 8). Clearly, the response of the caecal epithelial cells was significantly higher than that of the peritoneal mesothelial cells after the control abrasion. This may be the result of much lower gene expression in caecal samples from intact or sham-operated mice compared with peritoneal mesothelial cells from the same mice.

Three different groups of genes were identified: tPA, PAI-1, and fibrinogen α. TIMP-2 increased 20–60 times after abrasion (Figure 8a–d); fibrinogens β and γ and TGF-β increased 10 times (Figure 8e–g); MMP-9, 12 and TIMP-1 increased only 3–6 times (Figure 8h–j). The application of both types of PVP-Alg-based barriers (gel and film) effectively decreased the expression of multiple genes.

The calculated relative gene expression showed that the change in the expression of only three genes was associated with the protection induced by the PVP-Alg gel and film and, particularly, TIMP-2, fibrinogen γ, and MMP-12 (Figure 8, blue arrows). At the same time, enhanced fibrinogen γ and MMP-9 gene expressions led to an increase in the formation of adhesions in the PVP-Alg-Ca film groups (Figure 8, red arrow).

Among them, MMP-12 simply shows the involvement of macrophages, which is expected. It should be noted that, in the PVP-Alg gel group, it is produced by the macrophages accumulated in the granular tissue, while in the PVP-Alg-Ca film group, the macrophage pool runs out.

**Figure 9:**
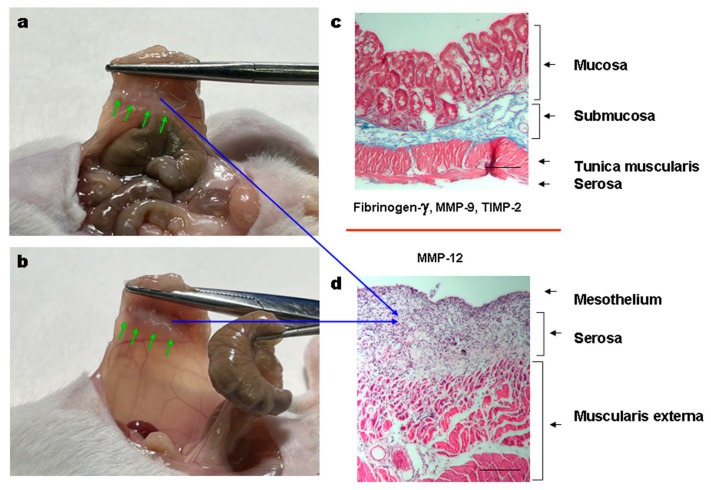
Overview and structure of the adhesions. (**a**,**b**): Adhesions in the PVP-Alg-Ca film group (**a**) and residual adhesion in the PVP-Alg gel group (**b**). (**c**,**d**) Histological image of adhesion sites produced by caecum (**c**) and peritoneal wall (**d**) cells. Scale bar—30 µm.

Our results demonstrated that fibrinogen γ likely plays a major role in the formation of adhesions. An increase in TIMP-2 and MMP-9 expressions in the PVP-Alg-Ca group can be indicative of the system’s ongoing efforts to eliminate adhesions. The residual adhesions can be observed in both the PVP-Alg and PVP-Alg-Ca groups (Figure 9a,b, green arrows). They are formed on the peritoneal wall and consist of connective tissue infiltrated by fibroblasts and macrophages (Figure 9c,d, blue arrows).

To summarize, the obtained results demonstrated that both the PVP-Alg and PVP-Alg-Ca films suppress multiple gene expression. However, the failure of the homeostatic system to degrade the insoluble PVP-Alg-Ca film prevented the elimination of the fibrous adhesions and the production of fibrinogen γ, MMP-9, and TIMP-2. This means that the ability of the barrier material to be timely removed is an important characteristic of the biopolymer.

## 4. Discussion

Fibrinolysis is one of the most important protective and adaptive systems of the body, providing hemostasis. The role of fibrinolysis in the formation and reformation of adhesions is to destroy fibrin clots formed during the healing process. The main active element in the process of fibrinolysis is plasmin. Plasminogen is transformed in plasmin with the participation of tissue-type plasminogen activator (tPA) and urokinase-type plasminogen activator (uPA). Plasmin causes fibrin degradation [38]. Both the plasminogen activators equally destroy fibrin clots, but the t-PA is more specific for fibrin, acting predominantly on fibrin-bound plasminogen. Plasminogen activator inhibitors (PAIs) form inactive complexes with t-PA and the u-PA [39]. The PAI-1 is a serine protease inhibitor protein secreted primarily by endothelial cells and peritoneal mesothelial cells. Endothelial cells, monocytes, macrophages, and fibroblasts all produce both types of PAIs [40]. The balance in the fibrinolysis changes during surgical interventions [41]. In this work, when using PVP-Alg-Ca films, there was a significant increase in the expression of PAI-1 in the cecum and abdominal wall cells in comparison with the intact mice. An increase in PAI-1 expression was also observed in the abdominal wall compared with mice operated on without anti-adhesive materials (the control). This was accompanied by extensive adhesions in the abdominal cavity, confirming the role of excess PAI-1 in suppressing fibrinolytic activity. However, the fibrinolytic activity is not always reduced because of a decrease in the expression of the tPA gene and an increase in PAI-1. In a number of studies, an increase in both the expression levels of PAI-1 and tPA was registered during the analysis of peritoneal fibrinolytic responses [41]. The analysis of peritoneal biopsies in humans taken at the beginning and the end of the operation showed an increase in tPA expression, while the PAI-1 expression index did not change [42]. When using the soluble PVP-Alg gels and films, the adhesions did not form, as compared with the control and PVP-Alg-Ca film group. Both types of PVP-Alg materials (gel and film) significantly reduced the increase in both tPA and PAI-1 occurring in the caecal tissue. This means that the PVP-Alg barrier materials effectively blocked the fibrinolytic system’s activation. Other genes, such as fibrinogen γ, MMP-9, and MMP-12, may be involved in the non-protective effects of PVP-Alg-Ca films. The protective effects of both barrier material types were found in TGF-β and fibrinogen α gene expressions.

Transforming factors take part in tissue reparation and adhesion formation [43,44]. However, the PVP-Alg material did not reduce the TGF-β gene expression’s ability to be protective at the same time, whereas the insoluble PVP-Alg-Ca films increased its effect, confirming the role of TGF-β in adhesion formation.

It is known that plasmin activates MMPs [45,46]. The proteolytic activity of MMPs is partially regulated by physiological inhibitors, the tissue inhibitors of MMPs (TIMPs). MMPs and TIMPs are the key regulators of cell migration; inflammation; angiogenesis; and, most importantly, the degradation of the extracellular matrix. Twenty genes (MMPs, collagenase, tropoelastin, collagen I) and four tested TIMPs are responsible for the formation and degradation of the extracellular matrix. However, only MMP-9, MMP-12, TIMP-1, and TIMP-2 were highly expressed in the samples of the caecum from the control mice. The major difference between the PVP-Alg gels and PVP-Alg-Ca films was the activation of fibrinogen γ and MMP-9, with MMP-12 gene expression only. It can be hypothesized that despite the protective effect of PVP-Alg-Ca films on the fibrinolytic system, the presence of insoluble PVP-Alg-Ca film in the peritoneal cavity can stimulate a pathogenic response. This is manifested by fibrinogen γ and MMP-9 production in an attempt to encapsulate the film and via the activation of macrophages that are able to remove it. MMP-12 is a macrophage metalloelastase or a macrophage elastase.

To summarize, PVP-Alg, being soluble, is eliminated by macrophages, as with other similar exogenous materials [47]. This fact is decisive in preventing the formation of adhesions. Crosslinking alginate makes it impossible to disintegrate the PVP-Alg-Ca film in the same way. Fibrinogen γ production and fibrosis occur despite the low adhesion of fibroblasts to the surface of the PVP-Alg-Ca material (Figure 2e). This is likely due to the absence of specific mechanisms of decomposition in structures such as crosslinked Alg. Crosslinking Alg might be subjected to bioresorption with the promotion of bone tissue de novo [48] via osteoclast activity—that is, specific bone tissue cells sensitive to calcium ions [49]. However, this only applies to bone tissue. In other cases, as in this work, crosslinked Alg did not undergo bioresorption [50].

Previously, the authors of [28] studied the anti-adhesion effects of barriers based on carboxymethylcellulose (CMC) and chitosan in a murine model. It was shown that the best result was achieved using materials based on CMC, preventing adhesion formation by 87%, though chitosan did not prevent adhesion formation. Considering that modern commercial anti-adhesion barriers are based on CMC and chitosan, the PVP-Alg material proposed in the present work looks promising, preventing the formation of adhesions by 95–100% (tested on a mouse model).

It should be taken into account that the results reported in the present work were obtained from mice, and further tests should be performed first in larger animals and, subsequently, humans. This is because the anti-adhesion effect of the material also depends on other factors, such as, for example, on its interaction with the biological environment, the size and severity of the injury, the size of the animal and its metabolism, etc. In particular, in an experiment on rabbits, non-crosslinked hyaluronic acid degraded too fast and ran out of the operating cavity, while the presence of iron-crosslinked hyaluronic acid was sufficient for a month until complete healing was achieved without adhesion formation [51,52]. These facts should be considered when planning further steps for the treatment of adhesions using the proposed PVP-Alg films.

## 5. Conclusions

The anti-adhesion activity of the soluble PVP-Alg and insoluble PVP-Alg-Ca materials was studied in vivo. Intraperitoneal surgery on mice was carried out, followed by histological and qPCR tests. Visual evaluation of adhesions, as well as the histology of the operated organs and an analysis of gene expression, allowed us to draw the following conclusions. The results of the gene expression analysis demonstrated that both types of materials effectively influenced the expression of several important genes responsible for adhesion formation. An increase in TGF-b1 expression in tissues was observed in both PVP-Alg and PVP-Alg-Ca films. However, in the case of the PVP-Alg-Ca film, the increase in the expression of this gene in the tissues of the caecum significantly exceeded its expression when using the PVP-Alg film. In this latter case, there was an increase in the expression of MMP-9 and -12 and TIMP-1 in the tissues of the caecum. MMP-9 and TIMP-1 also increased compared with the control animals. In the tissues of the abdominal wall, an increase in the expression of only MMP-12 was observed when compared with the intact and control animals. At the same time, the PVP-Alg-Ca films had no significant effect on the expression of MMP and TIMP genes. That is, the soluble PVP-Alg film induced the process of fibrinolysis, while the insoluble PVP-Alg-Ca film aggravated the inflammation and adhesion formation. Therefore, non-crosslinked soluble PVP-Alg films can be recommended as effective anti-adhesion barrier materials.

## Figures and Tables

**Table 1:** Assessment of the adhesive process.

Points	Description
0	No adhesion
1	Thin-film adhesion
2	Dense adhesion with small point attachment
3	More than one thin adhesion
4	Tight adhesion with flat attachment
5	A dense blood-supplied adhesion or more than one adhesion with a flat attachment

**Table 2:** List of primers.

No	Gene	Direct Primer	Reverse Primer
1	Actin-β	GGCTGTATTCCCCTCCATCG	CCAGTTGGTAACAATGCCATGT
2	tPA	TGCTGTGTGTACTGCTGCTT	TCTGCGTTGGCTCATCTCTG
3	PAI-1	AGTGTTTCAGCAGGTGGTCC	GACAAAGATGGCATCCGCAG
4	Fibrinogen α	GCCATCCCTAAACGCAGACA	AATCCTGGTTGGCTTCGTCA
5	Fibrinogen β	GAAAGTAGAACGGAGACCCCC	AGCGGAGCACACGAAGATT
6	Fibrinogen γ	CGGCTGGTGGATGAACAAATG	TGAAAATGAAGTGAGGTCCTGAAAG
7	MMP-9	GGGTCTAGGCCCAGAGGTAA	AGACACGCCCCTTGCTGA
8	MMP-12	TGCACTCTGCTGAAAGGAGTC	TGAGTTGTCCAGTTGCCCAG
9	TIMP-1	GGACCTGGTCATAAGGGCTA	GGCATATCCACAGAGGCTTT
10	TIMP-2	TTCCGGGAATGACATCTATGG	GGGCCGTGTAGATAAACTCGAT
11	TGF-β1	ACTGGAGTTGTACGGCAGTG	GGGGCTGATCCCGTTGATTT

tPA—tissue plasminogen activator; PAI-1—plasminogen activator inhibitor 1; MMP—matrix metalloprotease; TIMP—tissue inhibitor of matrix metalloproteases; TGF-β1—transforming growth factor beta 1.

**Table 3:** Anti-adhesive effect of PVP-Alg barrier material.

Group	Number of Animals	Adhesion Score						The Median Score	Standard Deviation	*p*-Value vs. Control
		0	1	2	3	4	5			
		Animal distribution								
Control	5	1	0	0	0	4	0	4	1.79	
PVP-Alg gel	5	5	0	0	0	0	0	0	0	0.019
PVP-Alg film	8	7	1	0	0	0	0	0	0.35	0.011
PVP-Alg-Ca film	5	0	0	0	0	0	5	5	0	0.006

## Data Availability

The experimental data on the results reported in this manuscript are available upon reasonable request from the corresponding author.
